# Statin use and acute kidney injury among hospitalized chronic kidney disease patients: a retrospective cohort study

**DOI:** 10.3389/fmed.2025.1639130

**Published:** 2025-09-01

**Authors:** Ling-Er Tang, Da-Min Xu, Ling-Yi Xu, You-Lu Zhao, Yi-Dan Zhu, Ji-Cheng Lv, Li Yang, Xi-Zi Zheng

**Affiliations:** ^1^Renal Division, Department of Medicine, Institute of Nephrology, Peking University First Hospital, Peking University, Beijing, China; ^2^China Key Laboratory of Renal Disease, Ministry of Health of China, Beijing, China; ^3^Clinical Research Institute, Peking University, Beijing, China; ^4^Institute of Advanced Clinical Medicine, Peking University, Beijing, China

**Keywords:** acute kidney injury, statin, chronic kidney disease, mortality, atorvastatin

## Abstract

**Background:**

Chronic kidney disease (CKD) constitutes a substantial burden in terms of cardiovascular disease and acute kidney injury (AKI). While statins are recommended for their cardiovascular benefits in CKD patients, their impact on AKI remains inconclusive.

**Methods:**

A retrospective screening was conducted on all adult hospital admissions from January 1, 2018, to December 31, 2020, including patients with CKD. Statin exposure was defined as any prescription within 48 h of admission. Patients were monitored until death, discharge, or a maximum of 30 days. The primary outcome was in-hospital AKI, with in-hospital mortality as the secondary outcome.

**Results:**

In a cohort of 5,376 patients, the median age was 72 years; 3,184 (59.2%) were male, and 2,129 (39.6%) were statin users. In-hospital AKI was observed in 149 (7.0%) of statin users compared to 213 (6.6%) of non-users. Statin use was significantly associated with a reduced risk of in-hospital AKI [adjusted hazard ratio [aHR], 0.74; 95% confidence interval [CI], 0.56–0.96] and in-hospital mortality (aHR, 0.45; 95% CI, 0.24–0.88). These outcomes were consistent across subgroup analyses stratified by age, gender, baseline estimated glomerular filtration rate (eGFR), and cardiovascular disease (all *P* for interaction >0.05), as well as in sensitivity analyses excluding patients who discontinued statin therapy during hospitalization or initiated statin therapy post-baseline. Among atorvastatin users (63.4%, 1,350/2,129), only medium-dose atorvastatin was significantly associated with reduced risk of in-hospital AKI after full adjustment (aHR, 0.68; 95% CI, 0.49–0.95).

**Conclusions:**

Statin use may improve survival and reduced AKI risk in hospitalized patients with CKD, with atorvastatin showing particularly favorable renoprotective effects.

## 1 Introduction

Acute kidney injury (AKI), characterized by rapid kidney function decline, affects over 20% of hospitalized patients and presents a significant clinical burden (1). A well-established bidirectional relationship exists between AKI and chronic kidney disease (CKD) (2): CKD significantly increases AKI risk (3), while AKI exacerbates CKD progression through maladaptive tubular repair and persistent inflammatory responses (4). This vicious cycle highlights the critical need for AKI prevention in CKD populations.

Statins, a cornerstone of lipid management with well-established cardiovascular benefits in the general population (5, 6), are also recommended for mortality reduction in CKD patients (7). Evidence suggests potential renoprotective effects of statins through pleiotropic effects (8). However, real-world adherence to statin use in CKD remains suboptimal (9), likely due to clinician concerns regarding drug safety and efficacy in this vulnerable population who often exhibit decreased renal clearance, multiple comorbidities, and comedication.

Although clinical trials rarely report AKI as a statin-associated adverse effect (10), real-world observational studies suggest a potential increased AKI risk with statin use (11–13). In a large cohort study of 43,438 patients followed for up to 6.5 years found that statin users had a 30% higher likelihood of developing AKI compared to non-users (13). This discrepancy may reflect selection bias in trials, which often exclude patients at high risk of AKI (e.g., advanced CKD or acute illness). Currently, there is a paucity of data examining the impact of statins on AKI among CKD patients, with studies reporting both protective and harmful effects (12–14). Consequently, this retrospective study aims to assess the influence of statin use on in-hospital outcomes in CKD patients, with the primary outcome being in-hospital AKI and the secondary outcome being in-hospital mortality.

## 2 Methods

### 2.1 Study population

This retrospective observational cohort study was conducted at Peking University First Hospital, China. The study population comprised all adult patients admitted to the hospital between January 1, 2018, and December 31, 2020. Individuals with CKD at the time of admission were included in the study. CKD was defined by a baseline estimated glomerular filtration rate (eGFR) of < 60 ml/min/1.73 m^2^, as calculated using the CKD-EPI (Chronic Kidney Disease Epidemiology Collaboration) equation (15), based on the initial serum creatinine (SCr) measurement obtained within 48 h of admission. Patients were excluded if they (1) had a hospital stay of < 24 h; (2) had SCr detected less than two times during hospitalization; (3) had a baseline eGFR < 15 ml/min/1.73 m^2^ or on dialysis; (4) with admitting diagnosis of AKI; (5) developed AKI within 48 h of admission. Only the first admission that met the criteria was included in the event of readmissions.

### 2.2 Data collection

Patient data were systematically gathered through medical chart abstraction utilizing the institution's clinical data warehouse, which encompasses comprehensive inpatient information. The baseline was established as the first 48 h post-admission. We included demographic data, chronic comorbidities, admission department, surgery information, concomitant medications, and laboratory tests. Chronic comorbidities were identified based on admission diagnoses, employing the International Classification of Diseases, 10th Revision (ICD-10) codes. Concomitant medications were ascertained from prescription records. Patients were followed up until death or discharge or 30 days after admission, whichever came first. All SCr values were documented to identify AKI.

### 2.3 Statin exposure

Statin use was defined as at least one prescription of statins within 48 h of admission. Stain type and dose were classified by the first prescription. Statin intensity was classified into low, moderate, and high according to the American College of Cardiology/American Heart Association Guideline on the Management of Blood Cholesterol (16).

### 2.4 Clinical outcomes

The primary outcome is AKI, defined as an increase in SCr by 50% or greater within 7 days or 26.5 mmol/L within 48 h, according to Kidney Disease Improving Global Outcomes (KDIGO) criteria (17). The secondary outcome is in-hospital mortality, defined as all causes of death. Other outcomes included severe AKI defined as AKI stage 3 using the KDIGO criteria (17) and etiologies of AKI, which was classified into pre-renal, intra-renal, and post-renal by manual checking. Two clinicians independently reviewed electronic health records to determine the etiology of AKI, with discrepancies resolved by a third senior clinician. Initially, we planned to investigate the association between statin use and myopathic injury (defined as creatine kinase ≥4 × upper limit of normal). Nevertheless, the low observed incidence (0.7%, 36/5,376) provided insufficient statistical power for this analysis.

### 2.5 Statistical analysis

Categorical variables were expressed as frequencies, and Chi-square or Fisher exact test was used to compare groups. Whereas, continuous variables were expressed as medians (interquartile range), non-parametric tests were used for group comparisons.

We used COX proportional hazard models to estimate the hazard ratio (HR) of outcomes, adjusting for 24 prespecified covariates. Covariates were chosen based on clinical experience, related literature (11, 18), and accessibility, including age, gender, body mass index, ICU admission (defined as hospitalized in ICU or transferred to ICU within 48 h of admission), chronic comorbidities (diabetes, hypertension, cardiovascular disease, cerebrovascular disease, severe liver disease, malignancy, and inflammatory and autoimmune disease), concomitant medications (contrast, proton pump inhibitors, renin-angiotensin-aldosterone system inhibitors, beta-blockers, diuretics, non-steroidal anti-inflammatory drugs, nephrotoxic antibiotics and chemotherapy agents), and baseline laboratory tests (eGFR, hemoglobin, serum albumin, creatine kinase, and D-dimer). HRs and 95% confidence intervals (CI) were reported for each model. Patients were censored at discharge or end of follow-up. Schoenfeld residual test was used to test the proportional hazards assumption, and no violations were detected. Although propensity score matching was initially explored to control for confounding, we determined it to be inappropriate for our study due to fundamental baseline differences between groups that prevented satisfactory matching. Multiple imputations were used to address missing values.

We also conducted prespecified subgroup analyses, including age, gender, baseline eGFR, and cardiovascular disease, and considered a *P*-value < 0.05 as a significant interaction. We initially planned to evaluate the relationship between statin intensity and outcomes. However, given that most patients (1,958/2,129, 92.0%) received moderate-intensity statin, there was insufficient statistical power to explore this hypothesis. Statin types were classified into atorvastatin, rosuvastatin, and other statins. To evaluate the impact of statin dose on clinical outcomes, we divided atorvastatin, the most common type in the study population, into low-dose (< 20 mg/d), medium-dose (20 mg/d), and high-dose (>20 mg/d) since the majority of patients (76%) were prescribed medium-dose atorvastatin (20 mg/d), as recommended by Chinese guidelines (19).

We performed comprehensive sensitivity analyses to address two potential sources of bias: (1) statin initiation/discontinuation and (2) lipid-lowering effects. To mitigate the risk of misclassifying statin exposure due to treatment changes during the follow-up period, we implemented two sequential analyses. The initial analysis excluded individuals who discontinued statin use during follow-up, defining statin users as those who commenced statin therapy within 48 h of admission and maintained usage throughout the follow-up period. The subsequent analysis excluded individuals who initiated statin therapy after the baseline phase, defining non-users as those who did not use statins at any point during the follow-up. Furthermore, to ascertain whether the observed renoprotective effects were independent of cholesterol modulation, we conducted additional analyses adjusting for serum low-density lipoprotein cholesterol (LDL-C) levels. All analyses were performed using R software (R, version 4.2.1).

## 3 Results

### 3.1 Clinical characteristics

**Figure 1:**
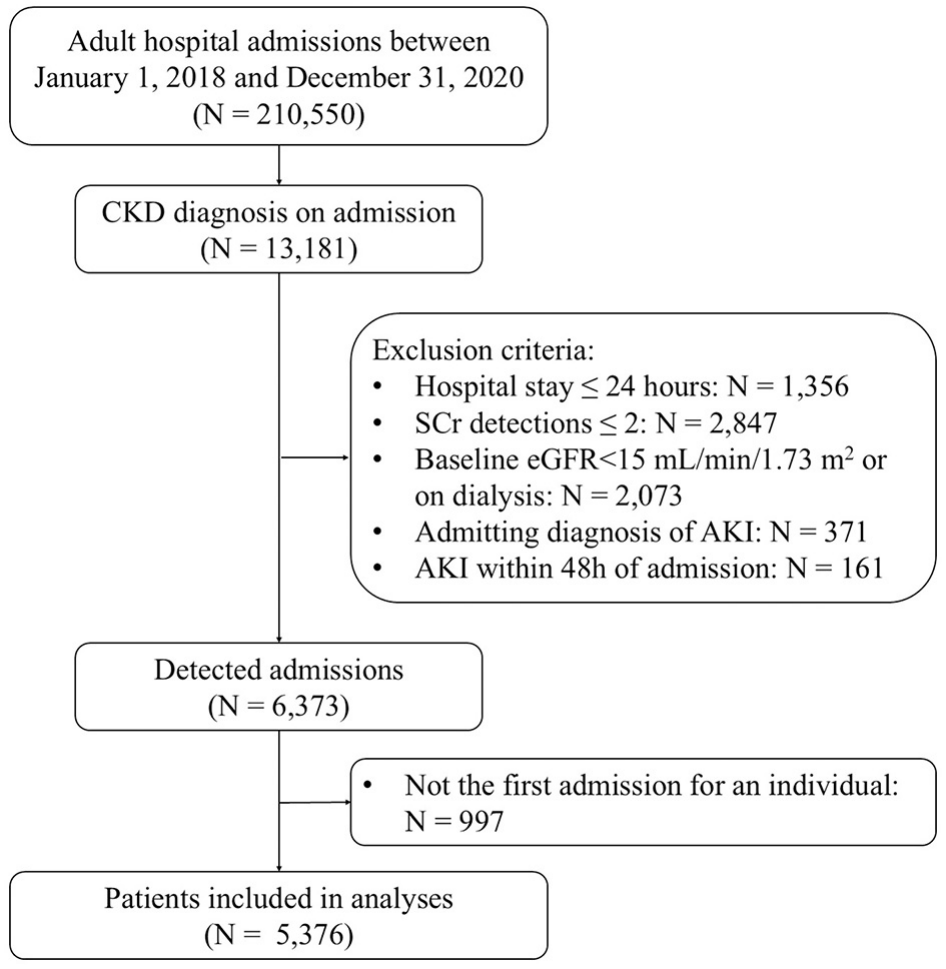
Flow diagram for this study selection. eGFR, estimated glomerular filtration rate; AKI, acute kidney injury.

A total of 5,376 CKD patients were included in this study (Figure 1). The median age was 72 years, and 59.2% were male. The median baseline eGFR was 47.9 ml/min/1.73 m^2^. Of these patients, 2,129 (39.6%) were prescribed statin within 48 h of admission (statin users). Compared with non-users, statin users were older, more likely to have an ICU admission, and had a higher comorbidity burden except for severe liver disease, malignancy as well as inflammatory and autoimmune disease. Likewise, more frequent use of concomitant medications was also found in statin users except for nephrotoxic antibiotics and chemotherapy agents. There was no significant difference in baseline eGFR between statin users and non-users (Table 1).

**Table 1:** Baseline characteristics of hospitalized CKD patients, categorized into statin users and non-users.

**Variables**	**All subjects (*N* = 5,376)**	**Statin user (*N* = 2,129)**	**Statin non-user (*N* = 3,247)**	***P*-value**
Age (y)	72.0 (61.0, 81.0)	75.0 (65.0, 82.0)	70.0 (59.0, 79.0)	<0.001
Male, *n* (%)	3,184 (59.2)	1,293 (60.7)	1,891 (58.2)	0.069
Body mass index (kg/m^2^)	25.0 (22.0,2 7.0)	25.0 (23.0, 28.0)	24.0 (22.0, 27.0)	<0.001
ICU admission^*^	462 (8.6)	306 (14.4)	156 (4.8)	<0.001
**Chronic comorbidity**, ***n*** **(%)**				
Dyslipidemia	1,879 (35.0)	1,493 (70.1)	386 (11.9)	<0.001
Hypertension	3,821 (71.1)	1,810 (85.0)	2,011 (61.9)	<0.001
Diabetes	1,965 (36.6)	1,090 (51.2)	875 (26.9)	<0.001
Cardiovascular disease	2,369 (44.1)	1,575 (74.0)	794 (24.5)	<0.001
Cerebrovascular disease	1,064 (19.8)	673 (31.6)	391 (12.0)	<0.001
Peripheral vascular disease	1,014 (18.9)	747 (35.1)	267 (8.2)	<0.001
Severe liver disease	89 (1.8)	9 (0.4)	80 (2.5)	<0.001
Malignancy	1,147 (21.3)	221 (10.4)	926 (28.5)	<0.001
Inflammatory and autoimmune disease	388 (7.2)	139 (6.5)	249 (7.7)	0.114
**Concomitant medication**, ***n*** **(%)**				
Contrast	284 (5.3)	119 (5.6)	165 (5.1)	0.416
Proton pump inhibitors	1,497 (27.8)	852 (40.0)	645 (19.9)	<0.001
Non-statin lipid-lowering agents	199 (3.7)	156 (7.3)	43 (1.3)	<0.001
RAAS inhibitor	1,753 (32.6)	1,118 (52.5)	635 (19.6)	<0.001
beta-blockers	1,380 (25.7)	962 (45.2)	418 (12.9)	<0.001
Diuretics	1,457 (27.1)	901 (42.3)	556 (17.1)	<0.001
NSAIDs	1,603 (29.8)	1,247 (58.6)	356 (11.0)	<0.001
Anticoagulants	1,974 (36.7)	1,313 (61.7)	661 (20.4)	<0.001
Nephrotoxic antibiotics	116 (2.2)	21 (1.0)	95 (2.9)	<0.001
Chemotherapy agents	185 (3.4)	34 (1.6)	151 (4.7)	<0.001
**Baseline laboratory tests**				
eGFR (ml/min. 1.73 m^2^)	47.9 (37.3, 54.8)	47.6 (37.7, 54.5)	48.0 (37.0, 55.1)	0.679
Hemoglobin (g/L)	124.0 (109.0, 136.0)	125.0 (111.0, 136.0)	124.0 (107.0, 137.0)	0.012
Serum albumin (g/L)	39.3 (35.2, 42.7)	38.7 (35.2, 41.8)	39.8 (35.2, 43.3)	<0.001
LDL-C (mmol/L)	2.4 (1.8, 3.1)	2.1 (1.6, 2.8)	2.6 (2.1, 3.2)	<0.001
ALT (U/L)	15.0 (11.0, 22.0)	16.0 (11.0, 23.0)	14.0 (10.0, 22.0)	<0.001
CK (U/L)	79.0 (51.0, 123.0)	85.0 (57.0, 135.0)	73.0 (47.0, 116.0)	<0.001
D-dimer (mg/L)	0.2 (0.1, 0.4)	0.2 (0.1, 0.4)	0.2 (0.1, 0.5)	0.019

* Hospitalized in ICU or transferred to ICU within 48 h of admission. Missing value: Body mass index (140), hemoglobin (41), AST (72), ALT (71), serum albumin (40), LDL-C (743), CK (1063), D-dimer (320). ICU, intensive care unit; RAAS inhibitors, renin-angiotensin-aldosterone system inhibitors; NSAIDs, non-steroidal anti-inflammatory drugs; eGFR, estimated glomerular filtration rate; LDL-C, low-density lipoprotein cholesterol; ALT, alanine transaminase; AST, aspartate transaminase; CK, creatine kinase.

### 3.2 Association between statin use and in-hospital AKI

During 9 (6, 14) days of follow-up, 362 in-hospital AKI events occurred. The incidence of in-hospital AKI was 7.0% (149/2,129) in statin users and 6.6% (213/3,247) in non-users. No significant association was found between statin use and AKI in the unadjusted model (HR, 1.08; 95% CI, 0.87–1.33). After adjusting for selected variables, statin use was associated with a decreased risk of AKI (aHR, 0.74; 95% CI, 0.56–0.96) (Table 2).

**Table 2:** Association between statin use with clinical outcomes in hospitalized CKD patients.

**Outcome**	**No. of patients with event (%)**		**Hazard ratio (95% confidence interval)**	
	**Statin user**	**Statin non-user**	**Unadjusted**	**Adjusted^a^**
**Primary outcome**				
Acute kidney injury	149 (7.0)	213 (6.6)	1.08 (0.87, 1.33)	0.74 (0.56, 0.96)
**Secondary outcome**				
In hospital mortality	17 (0.8)	56 (1.7)	0.47 (0.27, 0.81)	0.45 (0.24, 0.88)
**Other outcomes**				
Severe AKI	20 (0.9)	57 (1.8)	0.54 (0.32, 0.89)	0.52 (0.28, 0.97)
Pre-renal AKI	88 (4.1)	109 (3.4)	1.25 (0.94, 1.65)	0.67 (0.47, 0.97)
Intra-renal AKI	58 (2.7)	90 (2.8)	1.1 (0.79, 1.53)	0.95 (0.62, 1.47)
Post-renal AKI	3 (0.1)	14 (0.4)	0.32 (0.09, 1.13)	0.80 (0.23, 2.80)

a Adjusted for: age, gender, body mass index, ICU admission, chronic comorbidity (hypertension, diabetes, cardiovascular disease, cerebrovascular disease, severe liver disease, malignancy, inflammatory and autoimmune disease), medication (contrast, proton pump inhibitor, renin-angiotensin-aldosterone system inhibitors, beta-blockers, diuretics, non-steroidal anti-inflammatory drugs, nephrotoxic antibiotics, chemotherapy agents), laboratory tests (hemoglobin, serum albumin, eGFR, creatine kinase, D-dimer).

Among the 2,129 statin users, atorvastatin was the most prevalent (63.4%, 1,350/2,129), followed by rosuvastatin (21.3%, 453/2,129) and other statins (15.3%, 326/2,129). The median daily dosages were 20 mg for atorvastatin and 10 mg for rosuvastatin. As for the association between different types of statins and in-hospital AKI, compared with non-users, a significant reduction in in-hospital AKI was only observed in atorvastatin users after adjusting for all covariates (aHR 0.72; 95% CI 0.53–0.97) (Table 3). In the subgroup of atorvastatin users, only medium-dose of atorvastatin was significantly associated with decreased AKI risk (aHR 0.68, 95% CI 0.49–0.95) after full adjustment (Supplementary Table 1).

**Table 3:** Association of different types of statins with primary and secondary outcome.

**Subgroups**	**Acute kidney injury**			**In hospital mortality**		
	**Events (%)**	**Unadjusted HR (95%CI)**	**Adjusted HR (95%CI)^a^**	**Events (%)**	**Unadjusted HR (95%CI)**	**Adjusted HR (95%CI)^a^**
Non-users (*N* = 3,247)	213 (6.6)	Reference	Reference	56 (1.7)	Reference	Reference
Atorvastatin users (*N* = 1,350)	103 (7.6)	1.18 (0.93, 1.49)	0.72 (0.53, 0.97)	12 (0.9)	0.53 (0.28, 0.98)	0.52 (0.25, 1.09)
Rosuvastatin users (*N* = 453)	24 (5.3)	0.80 (0.52, 1.22)	0.62 (0.38, 1.01)	4 (0.9)	0.51 (0.18, 1.40)	0.54 (0.18, 1.62)
Other statin users (*N* = 326)	22 (6.7)	1.06 (0.68, 1.64)	1.01 (0.62, 1.65)	1 (0.3)	0.19 (0.03, 1.34)	0.21 (0.03, 1.61)

a Adjusted for: age, gender, body mass index, ICU admission, chronic comorbidity (hypertension, diabetes, cardiovascular disease, cerebrovascular disease, severe liver disease, malignancy, inflammatory and autoimmune disease), medication (contrast, proton pump inhibitor, renin-angiotensin-aldosterone system inhibitors, beta-blockers, diuretics, non-steroidal anti-inflammatory drugs, nephrotoxic antibiotics, chemotherapy agents), laboratory tests (hemoglobin, serum albumin, eGFR, creatine kinase, D-dimer).

The associations of statin use with AKI were broadly similar among subgroups classified by age, gender, baseline eGFR, and cardiovascular disease (all *P* for heterogeneity > 0.05) (Supplementary Figure 1). In sensitivity analysis (Supplementary Table 2), the association between statin use and AKI remained consistent when excluding patients who discontinued statin during hospitalization (aHR, 0.68, 95%CI 0.51–0.90) and when further excluding those who initiated statin after the baseline phase (aHR 0.70, 95%CI 0.52–0.95). The results remained consistent after further adjusting for LDL-C (aHR 0.72; 95% CI 0.53–0.98).

### 3.3 Association between statin use and in-hospital mortality

During 9 (6, 15) days of follow-up, a total of 73 patients died. Statin users had significantly lower in-hospital mortality than non-users (0.8% vs. 1.7%, *P* < 0.001). As shown in Table 2, a significant association between statin use and in-hospital mortality was observed in the unadjusted model (HR, 0.47; 95% CI, 0.27–0.81). After adjusting for potential confounders, the association was enhanced and remained statistically significant (aHR 0.45, 95% CI 0.24–0.88).

Regarding statin types and in-hospital mortality, in-hospital mortality rates were 0.9% (12/1,350) for atorvastatin, 0.9% (4/453) for rosuvastatin, and 0.3% (1/326) for other statins. Compared with non-users, only atorvastatin showed a reduction trend in in-hospital mortality, although this association was not statistically significant after full adjustment (Table 3).

Results from the subgroup analysis were consistent with the main results as the associations of statin use with in-hospital mortality were broadly similar among subgroups classified by age, gender, baseline eGFR, and cardiovascular disease (all *P* for heterogeneity > 0.05) (Supplementary Figure 2). In sensitivity analysis, statin use remained to be associated with decreased in-hospital mortality when we redefined statin users (aHR 0.29, 95%CI 0.13–0.63) and further redefined non-statin users (aHR 0.24, 95%CI 0.10–0.53) (Supplementary Table 2).

### 3.4 Association between statin use and other outcomes

As shown in Table 2, a total of 77 patients developed severe AKI. Statin use was associated with a decreased risk of severe AKI in the unadjusted model (HR 0.54, 95% CI 0.32–0.89) and adjusted model (aHR 0.52, 95% CI 0.28–0.97). As for different etiologies of AKI, statin use was associated with a statistically significant lower risk of pre-renal AKI (aHR 0.67, 95% CI 0.47–0.97), whereas the protective effect against intra-renal AKI (aHR 0.95, 95% CI 0.62–1.47) and post-renal AKI (aHR 0.80, 95% CI 0.23–2.80) was not significant.

## 4 Discussion

In this retrospective study of 5,376 hospitalized CKD patients, we found statin use was associated with a 26% reduction in in-hospital AKI and a 55% reduction in in-hospital mortality. These findings remained consistent in subgroup analyses stratified by age, gender, baseline eGFR, and cardiovascular disease.

The cardiovascular and survival benefits of statins have been conclusively demonstrated in both the general population (20) and non-dialysis CKD patients (21). *Post-hoc* analysis of the JUPITER trial demonstrated significantly reduced all-cause mortality with statin use in CKD patients (eGFR < 60 mL/min/1.73 m^2^; HR 0.56, 95% CI 0.37–0.85) (22), consistent with findings from another trial in moderate CKD (eGFR 30- < 60 mL/min/1.73 m^2^; HR 0.49, 95% CI 0.27–0.89) (23). While the SHARP study failed to demonstrate similar mortality benefits (24), this may reflect the inclusion of dialysis patients (33% of cohort) who exhibit attenuated statin responses (25). Our findings further strengthen the evidence for statin benefits in non-dialysis CKD, supporting KDIGO guideline recommendations (17). Despite these evidence-based recommendations, there is a reported under-prescription of statins in CKD populations, primarily due to concerns regarding drug safety within this specific group (9). AKI is a prevalent complication among CKD patients, particularly during hospitalizations for acute illnesses. In contrast to the well-established cardiovascular benefits, the effect of statins on AKI remains ambiguous (12–14). A large population-based cohort study involving 128,140 patients older than 66 years old revealed that the use of high-intensity (HR 1.17, 95% CI 1.09–1.26) and medium-intensity (HR 1.09, 95% CI 1.00–1.18) of statin use was associated with a reduced risk of AKI in CKD subgroup, respectively (26). Similarly, another meta-analysis of 2,313 patients with renal insufficiency found that statins significantly decreased the risk of contrast-induced AKI (RR 0.62, 95% CI 0.41–0.98) (27). However, contrasting evidence has emerged from a clinical trial of 1,922 patients undergoing cardiac surgery, where rosuvastatin resulted in a statistically significant 5.4 ± 1.9 percentage point increase in postoperative AKI (*P* = 0.005) (28). This finding aligns with a population-based cohort study of over 2 million patients in England and Wales (29), which reported adjusted HRs for AKI ranged from 1.50 to 2.19 across different statin types.

Variations in dosage and statin type may explain the inconsistent findings on statins and AKI risk. While high-dose statins are preferred for high-risk cardiovascular patients due to their superior efficacy (30), substantial evidence indicates that they may also increase AKI risk in a dose-dependent manner (11, 12, 29). Consequently, the KDIGO guidelines recommend lower statin doses for patients with advanced CKD (7). In our study, moderate-dose statins were predominately prescribed, likely reflecting clinicians' caution in this vulnerable population characterized by advanced age, reduced kidney function, comorbidities, and polypharmacy. This may account for the observed protective effect of statin use against AKI in hospitalized CKD patients. Regarding statin type, previous research has highlighted atorvastatin's superior renoprotection over rosuvastatin in CKD patients (31, 32), with a multicenter randomized controlled trial demonstrating significant lower AKI incidence (31) and a large-scale cohort study of nearly 1 million patients confirming fewer adverse kidney events (32). Therefore, although moderate-dose rosuvastatin (10 mg/day) was considered kidney safety (33), atorvastatin remains the preferred choice for patients with decreased kidney function. Our findings support this clinical preference, demonstrating predominance atorvastatin use and a more significant reduction trend in in-hospital AKI compared to rosuvastatin. These findings warrant validation through prospective, randomized studies to establish evidence-based recommendations for optimal statin selection and dosing in CKD populations.

Extensive research has demonstrated that statins exert renoprotection through pleiotropic mechanisms beyond lipid-lowering effects. In renal ischemia-reperfusion injury models, statin pretreatment alleviated kidney injury by reducing tubular necrosis, oxygen stress and lipid peroxidation (34). This protective effect was further supported by studies utilizing innovative drug delivery systems, where a ROS-responsive ceria nanoparticle-atorvastatin conjugate effectively mitigated oxidative stress, inflammation and tubular apoptosis *in vivo* and in sepsis-induced AKI models (35). Statins suppress TLR4 signaling to inhibit tubular cell pyroptosis while creating a favorable microenvironment for mesenchymal stem cells (36), which exert antioxidant effects via extracellular vesicles (37). Additionally, statins activate PPARγ-mediated PTEN upregulation, reducing renal inflammation and fibrosis by modulating immune cell and fibroblast infiltration (38, 39). These findings provide compelling mechanistic explanations for the observed clinical benefits in AKI, suggesting potential therapeutic applications for AKI prevention beyond their established cardiovascular indications in CKD patients.

This study has several limitations. First, the absence of outpatient data precluded the determination of the duration of preadmission statin use. Second, the observational design precludes the establishment of causality; however, multivariable adjustments and sensitivity analyses were conducted to mitigate potential biases, and the results remained robust. Third, using baseline eGFR to define CKD might capture patients with community-acquired AKI who fluctuate across the eGFR cutoff; we minimize this by excluding patients with admitting diagnosis of AKI or those who developed AKI within 48 h of admission. Fourth, the study lacked sufficient power to assess the impact of statin intensity. Finally, the absence of experimental data limited mechanistic exploration.

## 5 Conclusion

Statin use may not only improve survival but also protect against AKI in CKD patients. Atorvastatin demonstrates particularly promising renoprotective effects in the studied population. These findings highlight the need for future research to better characterize which patients derive the greatest benefit from statin use and to compare the efficacy of different statin types and dosages in this vulnerable population.

## Data Availability

The raw data supporting the conclusions of this article will be made available by the authors, upon reasonable request.

## Supplementary material

Supplementary Table 1 Association of dose of atorvastatin with primary and secondary outcome.

Supplementary Table 2 Sensitivity analysis of the association between statin use and primary and secondary outcome.

Supplementary Figure 1 Subgroup analysis in the association between statin use and acute kidney injury.

Supplementary Figure 2 Subgroup analysis in the association between statin use and in-hospital mortality.

## References

[1] Xu L Li C Li N Zhao L Zhu Z Zhang X. Incidence and prognosis of acute kidney injury versus acute kidney disease among 71 041 inpatients. Clin Kidney J. (2023) 16:1993–2002. 10.1093/ckj/sfad208 37915910 PMC10616447

[2] Zhu Z Hu J Chen Z Feng J Yang X Liang W. Transition of acute kidney injury to chronic kidney disease: role of metabolic reprogramming. Metabolism. (2022) 131:155194. 10.1016/j.metabol.2022.155194 35346693

[3] Zhang J Pang Q Zhou T Meng J Dong X Wang Z. Risk factors for acute kidney injury in COVID-19 patients: an updated systematic review and meta-analysis. Ren Fail. (2023) 45:2170809. 10.1080/0886022X.2023.2170809 37021610 PMC10081062

[4] Guzzi F Cirillo L Roperto RM Romagnani P Lazzeri E. Molecular mechanisms of the acute kidney injury to chronic kidney disease transition: an updated view. Int J Mol Sci. (2019) 20:E4941. 10.3390/ijms20194941 31590461 PMC6801733

[5] Pastori D Baratta F Di Rocco A Farcomeni A Del Ben M Angelico F. Statin use and mortality in atrial fibrillation: a systematic review and meta-analysis of 100,287 patients. Pharmacol Res. (2021) 165:105418. 10.1016/j.phrs.2021.105418 33450384

[6] Kokkinidis DG Arfaras-Melainis A Giannopoulos S Katsaros I Jawaid O Jonnalagadda AK. Statin therapy for reduction of cardiovascular and limb-related events in critical limb ischemia: a systematic review and meta-analysis. Vasc Med. (2020) 25:106–17. 10.1177/1358863X19894055 31964311

[7] Wanner C Tonelli M KDIGO. Clinical Practice Guideline for Lipid Management in CKD: summary of recommendation statements and clinical approach to the patient. Kidney Int. (2014) 85:1303–9. 10.1038/ki.2014.31 24552851

[8] Epstein M Campese VM. Pleiotropic effects of 3-hydroxy-3-methylglutaryl coenzyme a reductase inhibitors on renal function. Am J Kidney Dis. (2005) 45:2–14. 10.1053/j.ajkd.2004.08.040 15696439

[9] Kampmann JD Nybo M Brandt F Støvring H Damkier P Henriksen DP. Statin use before and after the KDIGO Lipids in chronic kidney disease guideline: a population-based interrupted time series analysis. Basic Clin Pharmacol Toxicol. (2022) 131:306–10. 10.1111/bcpt.13768 35762022 PMC9795967

[10] Tunnicliffe DJ Palmer SC Cashmore BA Saglimbene VM Krishnasamy R Lambert K. CoA reductase inhibitors (statins) for people with chronic kidney disease not requiring dialysis. Cochrane Database Syst Rev. (2023) 11:CD007784. 10.1002/14651858.CD007784.pub3 38018702 PMC10685396

[11] Coste J Karras A Rudnichi A Dray-Spira R Pouchot J Giral P. Statins for primary prevention of cardiovascular disease and the risk of acute kidney injury. Pharmacoepidemiol Drug Saf. (2019) 28:1583–90. 10.1002/pds.4898 31517431 PMC6916201

[12] Dormuth CR Hemmelgarn BR Paterson JM James MT Teare GF Raymond CB. Use of high potency statins and rates of admission for acute kidney injury: multicenter, retrospective observational analysis of administrative databases. BMJ. (2013) 346:f880. 10.1136/bmj.f880 23511950

[13] Acharya T Huang J Tringali S Frei CR Mortensen EM. Mansi IA. Statin use and the risk of kidney disease with long-term follow-up (84-year study). Am J Cardiol. (2016) 117:647–55. 10.1016/j.amjcard.2015.11.031 26742473

[14] Zhou X Dai J Xu X Wang Z Xu H Chen J. Comparative efficacy of statins for prevention of contrast-induced acute kidney injury in patients with chronic kidney disease: a network meta-analysis. Angiology. (2019) 70:305–16. 10.1177/0003319718801246 30261736

[15] Inker LA Eneanya ND Coresh J Tighiouart H Wang D Sang Y. New creatinine- and cystatin C-based equations to estimate GFR without race. N Engl J Med. (2021) 385:1737–49. 10.1056/NEJMoa2102953 34554658 PMC8822996

[16] Grundy SM Stone NJ Bailey AL Beam C Birtcher KK Blumenthal RS. 2018 AHA/ACC/AACVPR/AAPA/ABC/ACPM/ADA/AGS/APhA/ASPC/NLA/PCNA Guideline on the management of blood cholesterol: a report of the American College of Cardiology/American Heart Association Task Force on Clinical Practice Guidelines. Circulation. (2019) 139:e1082–143. 10.1161/CIR.0000000000000624 30586774 PMC7403606

[17] Khwaja A. KDIGO clinical practice guidelines for acute kidney injury. Nephron Clin Pract. (2012) 120:C179–84. 10.1159/000339789 22890468

[18] Tian Y Li X Wang Y Zhao W Wang C Gao Y. Association between preoperative statin exposure and acute kidney injury in adult patients undergoing cardiac surgery. J Cardiothorac Vasc Anesth. (2022) 36:1014–20. 10.1053/j.jvca.2021.07.031 34389211

[19] Wang Z Liu J Li J Wu N Lu G Chen Z. Chinese guidelines for lipid management. Chin Circul J. (2023) 38:237–71. 10.3760/cma.j.cn112148-20230119-00038 36925135

[20] Chou R Cantor A Dana T Wagner J Ahmed AY Fu R. Statin use for the primary prevention of cardiovascular disease in adults: updated evidence report and systematic review for the US preventive services task force. JAMA. (2022) 328:754–71. 10.1001/jama.2022.12138 35997724

[21] Barayev O Hawley CE Wellman H Gerlovin H Hsu W Paik JM. Statins, mortality, and major adverse cardiovascular events among US veterans with chronic kidney disease. JAMA Netw Open. (2023) 6:e2346373. 10.1001/jamanetworkopen.2023.46373 38055276 PMC10701610

[22] Ridker PM MacFadyen J Cressman M Glynn RJ. Efficacy of rosuvastatin among men and women with moderate chronic kidney disease and elevated high-sensitivity C-reactive protein a secondary analysis from the JUPITER (Justification for the Use of Statins in Prevention-an Intervention Trial Evaluating Rosuvastatin) trial. J Am Coll Cardiol. (2010) 55:1266–73. 10.1016/j.jacc.2010.01.020 20206456

[23] Nakamura H Mizuno K Ohashi Y Yoshida T Hirao K Uchida Y. Pravastatin and cardiovascular risk in moderate chronic kidney disease. Atherosclerosis. (2009) 206:512–7. 10.1016/j.atherosclerosis.2009.03.031 19423108

[24] Baigent C Landray MJ Reith C Emberson J Wheeler DC Tomson C. The effects of lowering LDL cholesterol with simvastatin plus ezetimibe in patients with chronic kidney disease (Study of Heart and Renal Protection): a randomised placebo-controlled trial. Lancet. (2011) 377:2181–92. 10.1016/j.ymed.2011.08.055 21663949 PMC3145073

[25] Herrington WG Emberson J Mihaylova B Blackwell L Reith C Solbu MD. Impact of renal function on the effects of LDL cholesterol lowering with statin-based regimens: a meta-analysis of individual participant data from 28 randomised trials. Lancet Diabetes Endocrinol. (2016) 4:829–39. 10.1016/S2213-8587(16)30156-5 27477773

[26] Tonelli M Lloyd AM Bello AK James MT Klarenbach SW McAlister FA. Statin use and the risk of acute kidney injury in older adults. BMC Nephrol. (2019) 20:103. 10.1186/s12882-019-1280-7 30909872 PMC6434639

[27] Cho Aj Lee Y-K Sohn SY. Beneficial effect of statin on preventing contrast-induced acute kidney injury in patients with renal insufficiency: a meta-analysis. Medicine. (2020) 99:e19473. 10.1097/MD.0000000000019473 32150109 PMC7478506

[28] Zheng Z Jayaram R Jiang L Emberson J Zhao Y Li Q. Perioperative rosuvastatin in cardiac surgery. New Engl J Med. (2016) 374:1744–53. 10.1056/NEJMoa1507750 27144849

[29] Hippisley-Cox J Coupland C. Unintended effects of statins in men and women in England and Wales: population based cohort study using the QResearch database. BMJ. (2010) 340:c2197. 10.1136/bmj.c2197 20488911 PMC2874131

[30] Sofat S Chen X Chowdhury MM Coughlin PA. Effects of statin therapy and dose on cardiovascular and limb outcomes in peripheral arterial disease: a systematic review and meta-analysis. Eur J Vasc Endovasc Surg. (2021) 62:450–61. 10.1016/j.ejvs.2021.05.025 34389230

[31] de Zeeuw D Anzalone DA Cain VA Cressman MD Heerspink HJL Molitoris BA. Renal effects of atorvastatin and rosuvastatin in patients with diabetes who have progressive renal disease (PLANET I): a randomised clinical trial. Lancet Diabetes Endocrinol. (2015) 3:181–90. 10.1016/S2213-8587(14)70246-3 25660356

[32] Shin J-I Fine DM Sang Y Surapaneni A Dunning SC Inker LA. Association of rosuvastatin use with risk of hematuria and proteinuria. J Am Soc Nephrol. (2022) 33:1767–77. 10.1681/ASN.2022020135 35853713 PMC9529194

[33] KDIGO 2024 clinical practice guideline for the evaluation and management of chronic kidney disease. Kidney Int. (2024) 105:S117–314. 10.1016/j.kint.2023.10.018 38490803

[34] Wu K Lei W Tian J Li H. Atorvastatin treatment attenuates renal injury in an experimental model of ischemia-reperfusion in rats. BMC Nephrol. (2014) 15:14. 10.1186/1471-2369-15-14 24423094 PMC3897885

[35] Yu H Jin F Liu D Shu G Wang X Qi J. ROS-responsive nano-drug delivery system combining mitochondria-targeting ceria nanoparticles with atorvastatin for acute kidney injury. Theranostics. (2020) 10:2342–57. 10.7150/thno.40395 32104507 PMC7019163

[36] Cai J Yu X Zhang B Zhang H Fang Y Liu S. Atorvastatin improves survival of implanted stem cells in a rat model of renal ischemia-reperfusion injury. Am J Nephrol. (2014) 39:466–75. 10.1159/000362623 24854145

[37] Allinson CS Pollock CA Chen X. Mesenchymal stem cells in the treatment of Acute Kidney Injury (AKI), Chronic Kidney Disease (CKD) and the AKI-to-CKD transition. Integr Med Nephrol Androl (2023) 10:e00014. 10.1097/IMNA-D-22-00014

[38] Teresi RE Planchon SM Waite KA Eng C. Regulation of the PTEN promoter by statins and SREBP. Hum Mol Genet. (2008) 17:919–28. 10.1093/hmg/ddm364 18065496

[39] Cao F Li Y Peng T Li Y Yang L Hu L. in kidney diseases: a potential therapeutic target in preventing AKI-to-CKD transition. Front Med. (2024) 11:1428995. 10.3389/fmed.2024.1428995 39165377 PMC11333338

