# Supplementary material for: Statin use and acute kidney injury among hospitalized chronic kidney disease patients: a retrospective cohort study
Source: Front Med (Lausanne). 2025 Sep 1;12:1639130. doi: 10.3389/fmed.2025.1639130 (PMC12433938; doi:10.3389/fmed.2025.1639130)
Supplement: Supplementary Table 1 — Association of dose of atorvastatin with primary and secondary outcome. [file Data_Sheet_1.zip › Supplemental Table 1.docx]

**Supplemental Table 1.** **Association of dose of atorvastatin with primary and secondary outcome.**

|  | **Acute kidney injury** | | | **In hospital mortality** | | |
| --- | --- | --- | --- | --- | --- | --- |
|  | Events (%) | Unadjusted  HR (95%CI) | Adjusted  HR (95%CI) ^a^ | Events (%) | Unadjusted  HR (95%CI) | Adjusted  HR (95%CI) ^a^ |
| Non-users (N = 3247) | 213(6.6) | Reference | Reference | 56(1.7) | Reference | Reference |
| Low-dose (N = 230) | 15(6.5) | 0.95(0.56,1.6) | 0.75(0.42,1.32) | 3(1.3) | 0.74(0.23,2.37) | 0.93(0.27,3.2) |
| Medium-dose (N = 1026) | 72(7.0) | 1.08(0.82,1.41) | 0.68(0.49,0.95) | 8(0.8) | 0.46(0.22,0.96) | 0.43(0.18,1.00) |
| High-dose (N = 94) | 16(17.0) | 3.42(2.05,5.68) | 1.58(0.83,3.01) | 1(1.1) | 0.72(0.1,5.21) | 0.65(0.08,5.64) |

^a^ Adjusted for: age, gender, body mass index, ICU admission, chronic comorbidity (hypertension, diabetes, cardiovascular disease, cerebrovascular disease, severe liver disease, malignancy, inflammatory and autoimmune disease), medication (contrast, proton pump inhibitor, renin-angiotensin-aldosterone system inhibitors, beta-blockers, diuretics, non-steroidal anti-inflammatory drugs, nephrotoxic antibiotics, chemotherapy agents), laboratory tests (hemoglobin, serum albumin, eGFR, creatine kinase, D-dimer
