# Supplementary material for: Statin use and acute kidney injury among hospitalized chronic kidney disease patients: a retrospective cohort study
Source: Front Med (Lausanne). 2025 Sep 1;12:1639130. doi: 10.3389/fmed.2025.1639130 (PMC12433938; doi:10.3389/fmed.2025.1639130)
Supplement: Supplementary Table 1 — Association of dose of atorvastatin with primary and secondary outcome. [file Data_Sheet_1.zip › Supplemental Table 2.docx]

Supplemental Table 2. Sensitivity analysis of the association between statin use and primary and secondary outcome

| **Sensitivity analysis** | **Acute kidney injury** | | | | **In hospital mortality** | | | |
| --- | --- | --- | --- | --- | --- | --- | --- | --- |
|  | Statin users n (%) | Statin non-users n (%) | Unadjusted  HR (95% CI) | Adjusted ^c^  HR (95% CI) | Statin users n (%) | Statin non-users n (%) | Unadjusted  HR (95% CI) | Adjusted ^c^  HR (95% CI) |
| 1a ^a^ | 129(6.4) | 213(6.6) | 1.00(0.8,1.24) | 0.68(0.51,0.90) | 10(0.5) | 56(1.7) | 0.30(0.16,0.60) | 0.29(0.13,0.63) |
| 1b ^b^ | 129(6.4) | 177(6.0) | 1.08(0.86,1.35) | 0.70(0.52,0.95) | 10(0.5) | 54(1.8) | 0.28(0.14,0.55) | 0.24(0.10,0.53) |

^a^ Statin users were defined as those who take statins within 48 hours of admission and continue using it during hospitalization.

^b^ Statin users were defined as those who take statins within 48 hours of admission and continue using it during hospitalization. Statin non-users were defined as those who never take statin during hospitalization.

^c^ Adjusted for: age, gender, body mass index, ICU admission, chronic comorbidity (hypertension, diabetes, cardiovascular disease, cerebrovascular disease, severe liver disease, malignancy, inflammatory and autoimmune disease), medication (contrast, proton pump inhibitor, renin-angiotensin-aldosterone system inhibitors, beta-blockers, diuretics, non-steroidal anti-inflammatory drugs, nephrotoxic antibiotics, chemotherapy agents), laboratory tests (hemoglobin, serum albumin, eGFR, creatine kinase, D-dimer)
