# Supplementary figures and images for: Statin use and acute kidney injury among hospitalized chronic kidney disease patients: a retrospective cohort study
Source: Front Med (Lausanne). 2025 Sep 1;12:1639130. doi: 10.3389/fmed.2025.1639130 (PMC12433938; doi:10.3389/fmed.2025.1639130)

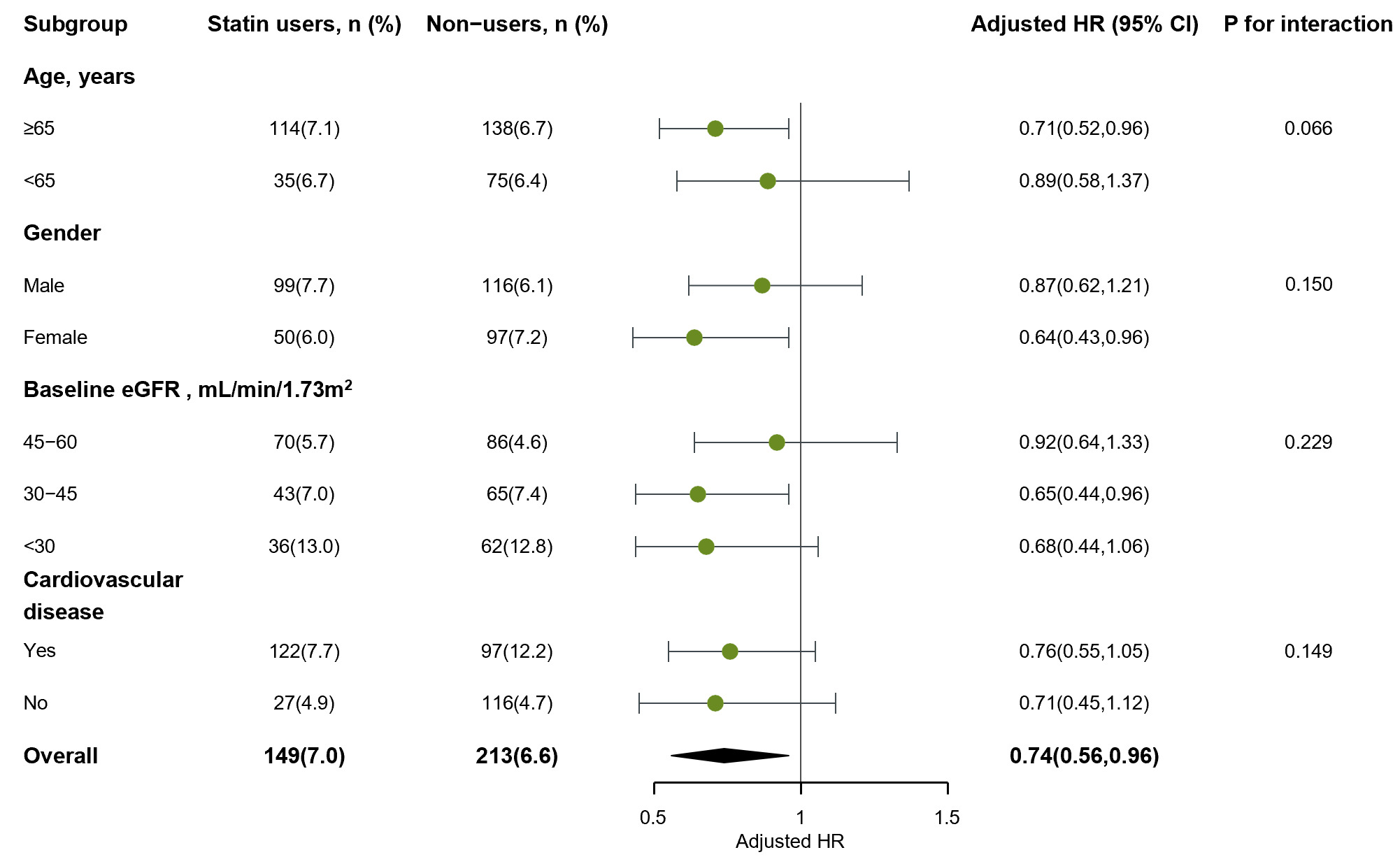

Supplement: Supplementary Table 1 — Association of dose of atorvastatin with primary and secondary outcome. [file Data_Sheet_1.zip › Sup Figure 1.jpg]

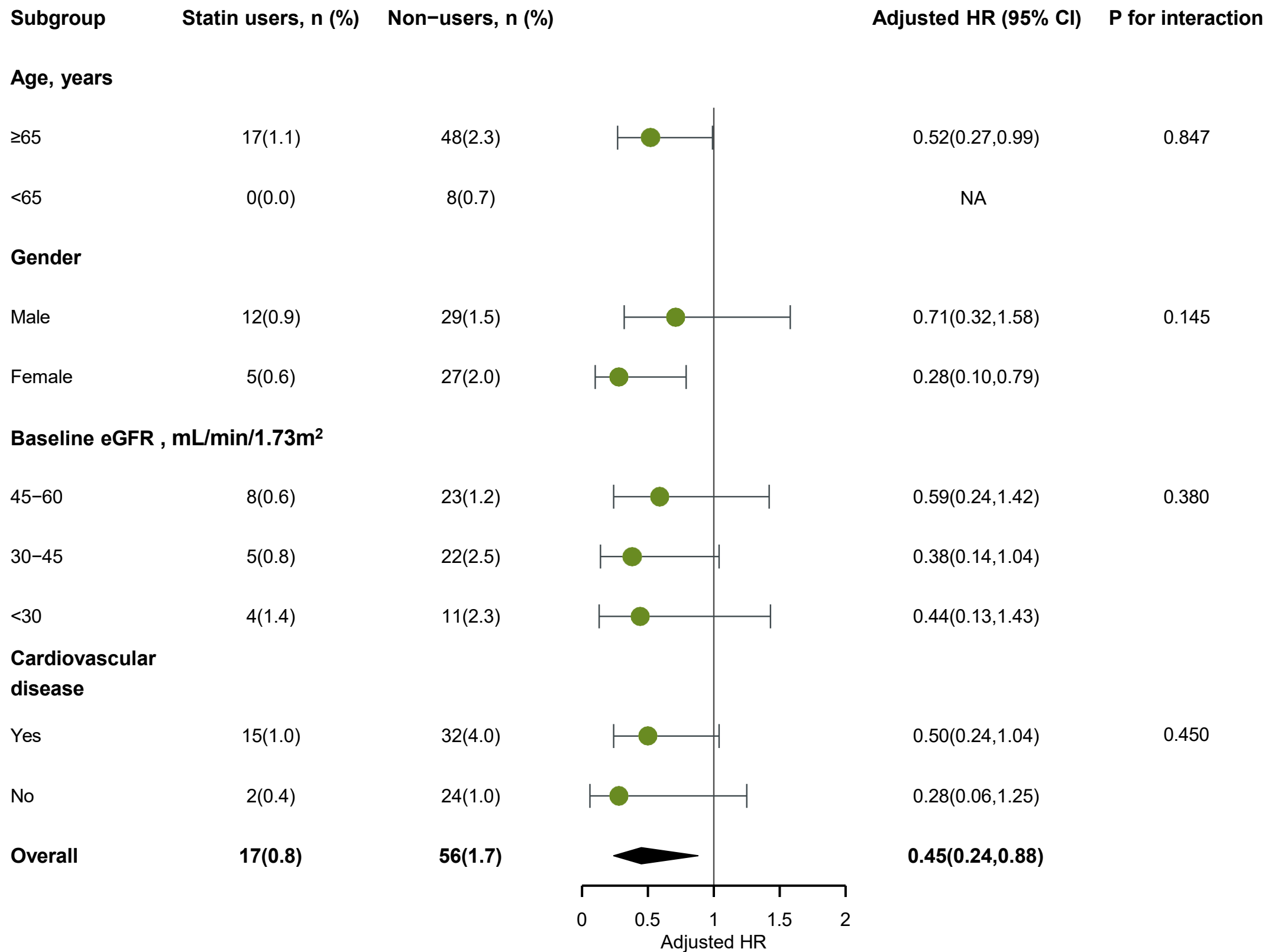

Supplement: Supplementary Table 1 — Association of dose of atorvastatin with primary and secondary outcome. [file Data_Sheet_1.zip › Sup Figure 2.pdf]
